# P-231. Association Between the Use of the Pneumonia Panel and Clinical Outcomes in Patients with Ventilator-Associated Pneumonia in a Peruvian Hospital

**DOI:** 10.1093/ofid/ofae631.435

**Published:** 2025-01-29

**Authors:** Luis M Salas Guzmán, Paola L Rondan, Jaime T Martínez, Alvaro Taype-Rondan, Laura Melissa Mori-Llontop

**Affiliations:** National Major University of San Marcos, Rímac, Lima, Peru; Universidad Nacional Mayor de San Marcos, Lima, Lima, Peru; Hospital de Lima Este Vitarte / Universidad Nacional Mayor de San Marcos, Lima, Lima, Peru; Universidad San Ignacio de Loyola, Lima, Lima, Peru; Universidad Peruana Cayetano Heredia, Lima, Lima, Peru

## Abstract

**Background:**

Ventilator-associated pneumonia (VAP) is a public health problem due to its high mortality in low-income countries. Therefore, the pneumonia panel emerges as a microbiological alternative due to its rapid response, allowing an early and targeted antibiotic treatment, which could reduce morbidity and mortality. The objective of the study was to describe the association between the use of the pneumonia panel versus culture alone and its clinical impact on patients with VAP in the intensive care unit (ICU).

Clinical characteristics and outcomes in patients with VAP in the ICU of a hospital in Peru, 2021-2022

**Figure:**
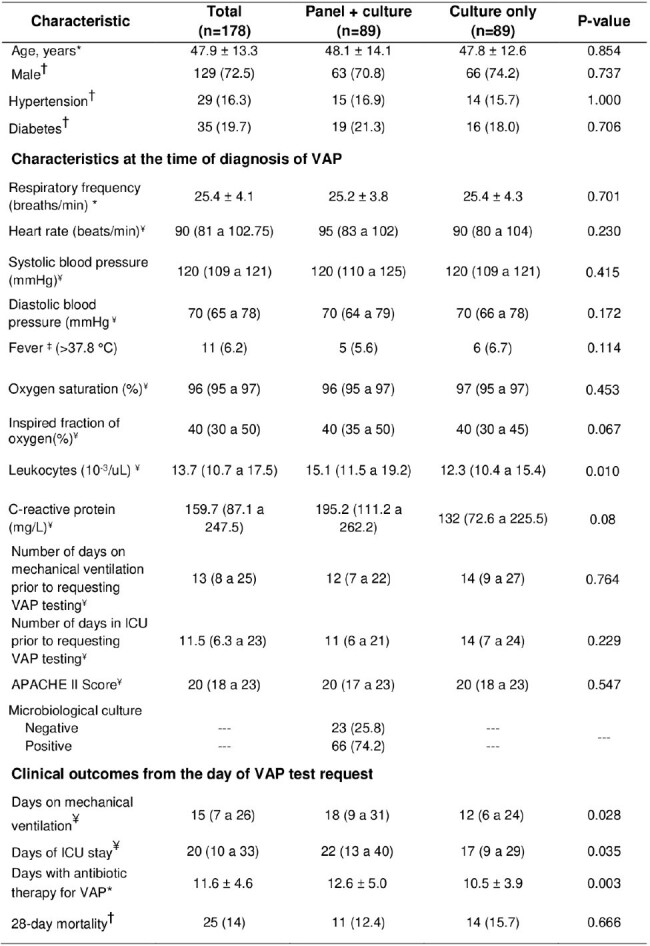

*Means ± standard deviations were used. Differences were evaluated using Student's t test.

†Frequencies were compared using the Chi-square test.

‡Frequencies were compared using Fisher's exact test.

¥Medians (25th and 75th percentiles) were used. Differences were evaluated using the equality of medians test.

**Methods:**

This retrospective cohort included patients diagnosed with VAP in the ICU of a hospital in Lima, Peru from May 2021 to February 2022. All patients diagnosed by panel + culture (n=89) were included, and 89 controls diagnosed only by culture were randomly assigned. Adjusted regressions were used to evaluate the association with clinical outcomes.

Isolated microorganisms and antimicrobial resistance genes in patients with VAP in the ICU of a hospital in Peru, 2021-2022

**Figure:**
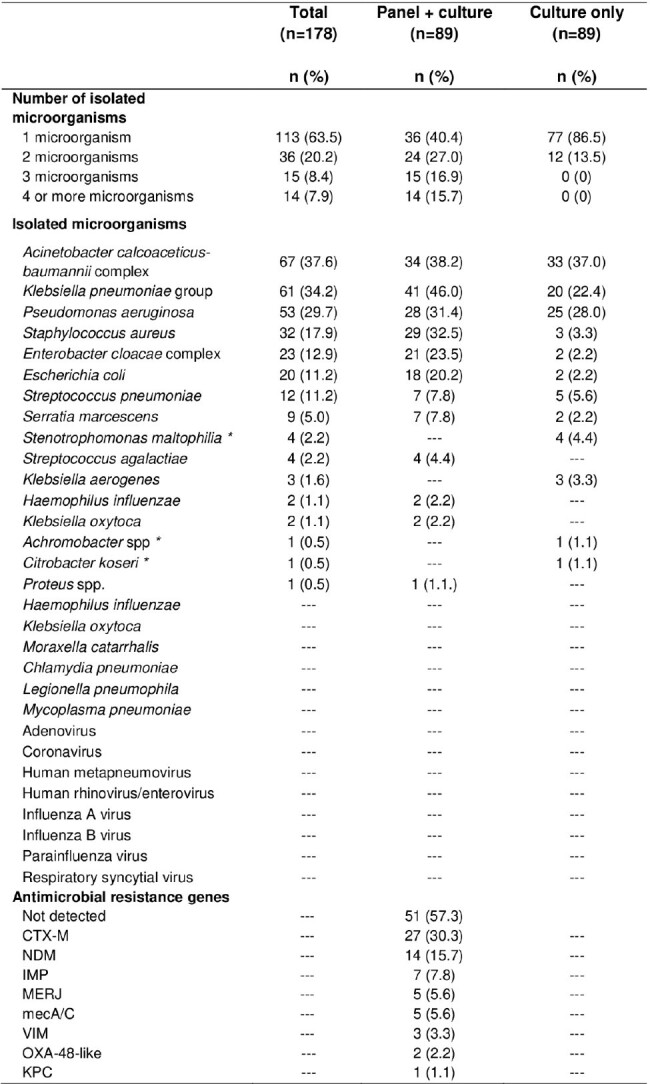

All microorganisms that can be detected by the FilmArray Plus pneumonia panel have been placed, and those that have been detected by culture have been added to the culture only group.

*The FilmArray Plus Pneumonia Panel does not detect microorganisms such as Stenotrophomonas maltophilia, Achromobacter spp and Citrobacter koseri.

**Results:**

The mean age was 47 years, 72.5% were men, 19.7% had diabetes and 16.3% had high blood pressure. The most identified microorganisms were: *Acinetobacter calcoaceticus-baumannii* complex (37.6%), *Klebsiella pneumoniae* group (34.2%), *Pseudomonas aeruginosa* (29.7%), *Staphylococcus aureus* (17.9%), *Enterobacter cloacae* complex (12.9%) and *Escherichia coli* (11.2%). The most frequent resistance genes were: CTX-M (30.3%) and NDM (15.7%).

When comparing the "panel + culture" and "culture only" groups, leukocyte counts (15.1*10^-3^/uL versus vs. 12.3*10^-3^/uL) and C-reactive protein level (195 mg/L versus 132 mg/L) were higher in the panel-use group. Then, adjusted analyses found no statistically significant differences in mortality (RR: 0.56, 95% CI: 0.25 to 1.27), days on mechanical ventilation (MD: 4.74, 95% CI: -0.76 to 10.23), or days in the ICU (MD: 4.94, 95% CI: -1.09 to 10.97). However, the "panel + culture" group had more days of antibiotic therapy (MD: 2.00, 95% CI: 0.45 to 3.55), as well as a greater number of isolated microorganisms.

Association between group and clinical outcomes in patients with VAP in the ICU of a hospital in Peru, 2021-2022

**Figure:**
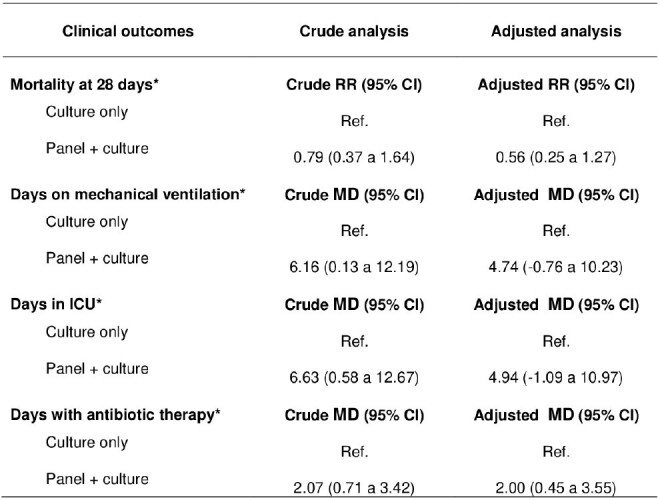

RR: Relative risk

MD: Mean Difference

The adjusted models were adjusted for diastolic blood pressure, fever, FiO2, leukocytes, and C-reactive protein (variables that had a p<0.20 association with group).

*The days were counted after the NAV test was requested.

**Conclusion:**

We found no differences in mortality, number of days in the ICU, and days on mechanical ventilation. However, there was evidence of a greater number of days with antibiotic therapy in the panel-use group.

Antibiotic treatment in patients during the VAP episode in the ICU of a hospital in Peru, 2021-2022

**Figure:**
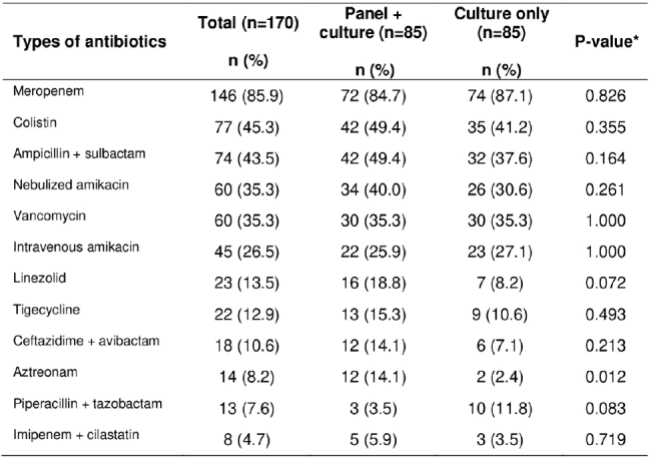

* Chi-square test was used, except for imipenem + cilastatin in which the Fisher's exact test was used.

**Disclosures:**

**All Authors**: No reported disclosures

